# Alcohol consumption and cardiovascular risk: a descriptive study in a psychiatric short stay unit

**DOI:** 10.1192/j.eurpsy.2023.1588

**Published:** 2023-07-19

**Authors:** C. González Navarro, I. Alonso Salas, L. Morado San segundo, A. López Fariña, A. Bilbao Idarraga, U. López Puentes, B. Samsó Martínez, R. F. Lopez Brokate, T. Ruiz de Azua Aspizua, E. M. Garnica de Cos, U. Ortega Pozas

**Affiliations:** 1 RED DE SALUD MENTAL DE BIZKAIA, ZAMUDIO, Spain

## Abstract

**Introduction:**

Patients with mental disorders have a decreased life expectancy, being the main reason the cardiovascular disease. An important proportion of patients present a comorbid drug consumption. Amongst drugs, alcohol is the most frequent, and it is associated with a higher cardiovascular risk. The metabolic syndrome is one of the most employed tools to assess cardiovascular risk.

**Objectives:**

- To describe the demographic characteristics of the patients with an active alcohol consumption that were admitted to the hospital during the period of study.

- To describe the prevalence of metabolic syndrome in the sample, according to the Adult Treatment Panel III (ATP-III) criteria.

**Methods:**

Retrospective observational study of three months duration. Data was collected from all patients admitted to the hospital during the period of study, with no specific exclusion criteria. Descriptive statistics were performed.

**Results:**

During the period of study 172 patients were admitted to the hospital (56.4% women and 43.6% men). A 44.8% presented alcohol consumption (25% sporadically, 6.4% weekly and 13.4% daily). Amongst women, 1% presented daily and 1% weekly consumption. Amongst men, 21.3% presented daily and 5.3% weekly consumption.

The prevalence of metabolic syndrome in the study sample was 29.11%. In the alcohol consumption group, the prevalence was 24.7% and differed according to the pattern of consumption: 43.5% in the daily consumption group, 27.3% in the weekly and 14% in the sporadically consumption group.

**Conclusions:**

On the one hand, in the sample of study a higher percentage of men present an active alcohol consumption, compared to women. It is remarkable the high percentage of daily alcohol consumption amongst men in our sample.

On the other hand, the prevalence of metabolic syndrome in our sample is similar to the one found in scientific literature regarding patients with mental disorders. It is noteworthy in our sample the increased prevalence of metabolic syndrome found in patients with a daily alcohol consumption, and a decreased prevalence in those with a sporadic pattern.

**Disclosure of Interest:**

None Declared

